# Reproducibility of Structural, Resting-State BOLD and DTI Data between Identical Scanners

**DOI:** 10.1371/journal.pone.0047684

**Published:** 2012-10-25

**Authors:** Lejian Huang, Xue Wang, Marwan N. Baliki, Lei Wang, A. Vania Apkarian, Todd B. Parrish

**Affiliations:** 1 Department of Physiology, Feinberg School of Medicine, Northwestern University, Chicago, Illinois, United States of America; 2 Department of Radiology, Feinberg School of Medicine, Northwestern University, Chicago, Illinois, United States of America; 3 Department of Psychiatry & Behavioral Sciences, Feinberg School of Medicine, Northwestern University, Chicago, Illinois, United States of America; 4 Departments of Anesthesia and Surgery, Feinberg School of Medicine, Northwestern University, Chicago, Illinois, United States of America; Institute of Psychology, Chinese Academy of Sciences, China

## Abstract

Increasingly, clinical trials based on brain imaging are adopting multiple sites/centers to increase their subject pool and to expedite the studies, and more longitudinal studies are using multiple imaging methods to assess structural and functional changes. Careful investigation of the test-retest reliability and image quality of inter- or intra- scanner neuroimaging measurements are critical in the design, statistical analysis and interpretation of results. We propose a framework and specific metrics to quantify the reproducibility and image quality for neuroimaging studies (structural, BOLD and Diffusion Tensor Imaging) collected across identical scanners and following a major hardware repair (gradient coil replacement). We achieved consistent measures for the proposed metrics: structural (mean volume in specific regions and stretch factor), functional (temporal Signal-to-Noise ratio), diffusion (mean Fractional Anisotropy and Mean Diffusivity in multiple regions). The proposed frame work of imaging metrics should be used to perform daily quality assurance testing and incorporated into multi-center studies.

## Introduction

MR brain imaging is a non-invasive tool with high spatial and temporal resolution that can be used to investigate brain function, and subtle structural changes related to learning, development, treatment, or as a result of a pathophysiologic condition. Standard (commonly used) MR imaging methods include high resolution structural scanning (T1 or T2 contrast) for brain morphometry changes, functional changes (BOLD) to study the dynamic changes in the brain related to a stimulus or in the resting condition, diffusion tensor imaging (DTI) and derived fractional anisotropy (FA) maps for monitoring white matter integrity and connectivity.

Increasingly clinical trials are adopting multiple sites/centers to increase their subject pool and to expedite the studies. Data sharing is another effective approach to increase the subject pool in populations that are difficult to recruit or require large numbers, eg. The Alzheimer’s Disease Neuroimaging Initiative (ADNI), Multidisciplinary Advances in Pelvic Pain (MAPP), and 1000 Functional Connectomes Project (fcon 1000). Longitudinal studies following the progression of disease have to consider the variability induced by software and hardware upgrades to the MRI scanner. Additional complications arise when incorporating scanners from different vendors, field strengths, software levels, and coil architecture. Careful investigation of the test-retest reliability and image quality of inter- or intra- scanner neuroimaging measurements are critical in the statistical analysis and interpretation of results. Previously, such studies have focused on a single imaging methodology specific metric: e.g. Cortical thickness based on T1 (Wonderlick et al., 2009), temporal Signal-to-Noise Ratio (Parrish et al., 2000) and FA values based on DTI (Vollmar et al, 2010).

In this paper, we propose a framework for quantifying the reproducibility and image quality for multimodal neuroimaging data (structural, BOLD and DTI) collected across identical scanners and following a major hardware repair (gradient coil replacement). The uniqueness of our study is (1) we examined the three standard brain imaging methods (T1, BOLD, DTI); (2) we examined the impact of a major hardware repair (gradient coil); and (3) we tested a battery of metrics for image quality and reliability assessment applicable to local and multi-center imaging trials.

## Methods

### Subjects and Experimental Design

Six healthy volunteers (five males, one female, mean age in years 26±4) participated in this study. The study was approved by the Northwestern University Institutional Review Boards. Written informed consent from all participants involved in the study was obtained. Within a one-month period all subjects were repeatedly scanned under the following three conditions: **condition 1**: scanner 1 (baseline); **condition 2**: scanner 1 (post gradient coil replacement); **condition 3**: scanner 2. Scanners 1 and 2 are identical Siemens 3.0 Tesla Trio Tim whole body systems, both equipped with a 32-channel head coil using VB17 software, as well as the same RF and magnetic shielding, and identical physical environment including power and chilled water. For each subject, **conditions** 2 and 3 were scanned back to back within an hour of each other and approximately 2 weeks after **condition** 1.

### Data Acquisition

T1 anatomical data were acquired following the ADNI protocol except with a 1 mm thickness using magnetization-prepared rapid acquisition with gradient echo (MPRAGE) with the following parameters: voxel size, 1 mm isotropic; TR/TE, 2300/2.97 ms; flip angle = 9^0^; in-plane acquisition matrix, 256×256; 176 1 mm slices. Resting state fMRI data were acquired using a gradient-echo echo-planar imaging sequence with the following parameters: voxel size, 1.72×1.72×3 mm; TR/TE, 2500/25 ms; flip angle = 80^0^; in-plane acquisition matrix, 120×128; Grappa factor of 2 with 24 reference lines, 40 slices, with 244 volumes. DTI data were acquired using the following acquisition parameters: voxel size, 2 mm isotropic; TR/TE, 9000/83 ms; flip angle = 90^0^; in-plane matrix resolution, 112×130; 72 slices; b = 1000 s/mm^2^. Diffusion was measured in 60 directions separated into seven groups by eight no-diffusion weighted volumes acquired for better registration and head motion correction.

### Data Analysis

Analyses were conducted using the FSL 4.1 (www.fmrib.ox.ac.uk/fsl/) [1] and MATLAB 7.9.0 on a clustered Linux system. To assess the reliability of the data across the above three conditions, specific imaging data based metrics including two metrics for the T1 image (volume of specific ROIs and stretch factor), one for resting state fMRI (temporal signal to noise ratio, tSNR, in specific ROIs) and two for the DTI data (mean fractional anisotropy and mean diffusivity in specific ROIs) were evaluated.

### Data Quality Control

To ensure the quality of the imaging data, motion for the subject during resting state fMRI and diffusion imaging were estimated. The maximum adjacent volume to volume motion for each subject is listed in Table 1 (middle and right columns) and all motion was less than 1 mm for the resting state fMRI and diffusion scans for all subjects under all three conditions. DTI images were also visually checked to ensure there was no slice drop-out due to excessive motion. Motion during the T1 scan often affects the background intensity anterior to head since the phase encoding direction is from anterior to posterior. To detect motion during the T1 scan, the ratio of background intensity between a region anterior to head (as sensitive measure to motion) and a region superior to eye (as reference), were calculated. Both regions contain 8000 voxels. A ratio larger than one raises concerns for motion and needs more careful visual inspection. As shown in Table 1 left column, except for three, all the other ratios are less than 1. The three T1 were inspected and there was some ghosting due to movement but the ghosting noise was insignificant compared to the signal in the brain.

**Table 1:** Motion parameters of six subjects across three conditions for T1, resting-state data, and DTI.

	T1 (Ratio Between anterior and superior ROIs)			Resting-state Data (Max motion (mm))			DTI (Max motion (mm))		
	Con1	Con2	Con3	Con1	Con2	Con3	Con1	Con2	Con3
Sub1	0.66	0.95	0.83	0.41	0.65	0.34	0.65	0.38	0.25
Sub2	0.84	1.39	0.93	0.20	0.54	0.30	0.71	0.27	0.30
Sub3	0.62	0.81	1.47	0.19	0.53	0.29	0.41	0.67	0.13
Sub4	0.72	0.74	0.64	0.09	0.34	0.14	0.09	0.78	0.37
Sub5	0.94	0.69	0.91	0.10	0.08	0.68	0.20	0.58	0.30
Sub6	1.13	0.93	1.00	0.08	0.16	0.19	0.43	0.24	0.22

Left three columns are the ratios between anterior and superior ROIs. Anterior ROI is defined as the region anterior to the skull but far from the level of eyes and its coordinate is within (90<x<109, 246<y<255, 186<z<225), totally 8000 voxels. Superior ROI is defined as the region superior to the skull and its coordinate is within (65<x<84, 156<y<195, 246<z<255), totally 8000 voxels. Middle three columns are the maximum displacement between adjacent volumes for resting-state data. Right three columns are the maximum displacement between volumes for the DTI data.

### Global Intensity Normalization

Due to inter-session differences in the physical state of the scanner hardware (e.g. coil loading), the interactions between the patient’s body with the scanner (e.g. positioning, different body sizes etc.) and different receiver settings [2], global image intensity varies from session to session. Therefore, the procedure of global intensity normalization was performed, in which each individual T1 image was multiplied by a scaling factor *G_i_*,
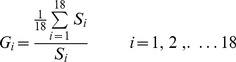
(1)where *S_i_* is the average intensity within the whole brain regions for the *i*th T1 image.

### Metric 1: T1 (ROIs Volume)

Volume of brain structures is often used to assess the status and progress of disease, aging, development etc. For a normal adult brain the volume should remain constant for a short period of time. The ROIs volume based on segmentation of the T1 anatomic data was used as a stability metric. The steps to estimate volume were as follows, Firstly, global intensity normalization was performed, then the brain was segmented using FIRST (part of FSL tools) [3]. The volume for the following 7 ROIs were estimated Thalamus (right), Putamen (right), Hippocampus (right), Amygdala (right), brain stem, white matter (WM) and cerebrospinal fluid (CSF).

### Metric 2: T1 (Stretch Factor)

The stretch factor describes the transformation required to move the native-space brain to the template space which is derived as the determinant of the affine transform matrix, similar to Atlas Scaling Factor [4]. The stretch factor is a good metric to quantify geometric distortions introduced in the morphometric measures. A stretch factor of 1 represents no change between individual space and template space. A stretch factor larger or smaller than 1 indicates expansion or contraction required to register each individual to the template respectively. Two templates were used for comparison; one was the 1 mm brain template from the Montreal Neurological Institute provided with FSL (MNI152_T1_1 mm). The other maintained the highest level of resolution by using a subject specific template acquired during condition 2. Using a 12-parameter affine transformation in FLIRT (part of FSL tools) [5], the individual subject’ T1 volume was co-registered to these two templates, and stretch factor (linear scaling) in the X, Y and Z directions was calculated and compared across the three conditions.

### Metric 3: Temporal Signal to Noise Ratio (tSNR) for Resting State fMRI

**Figure 1:**
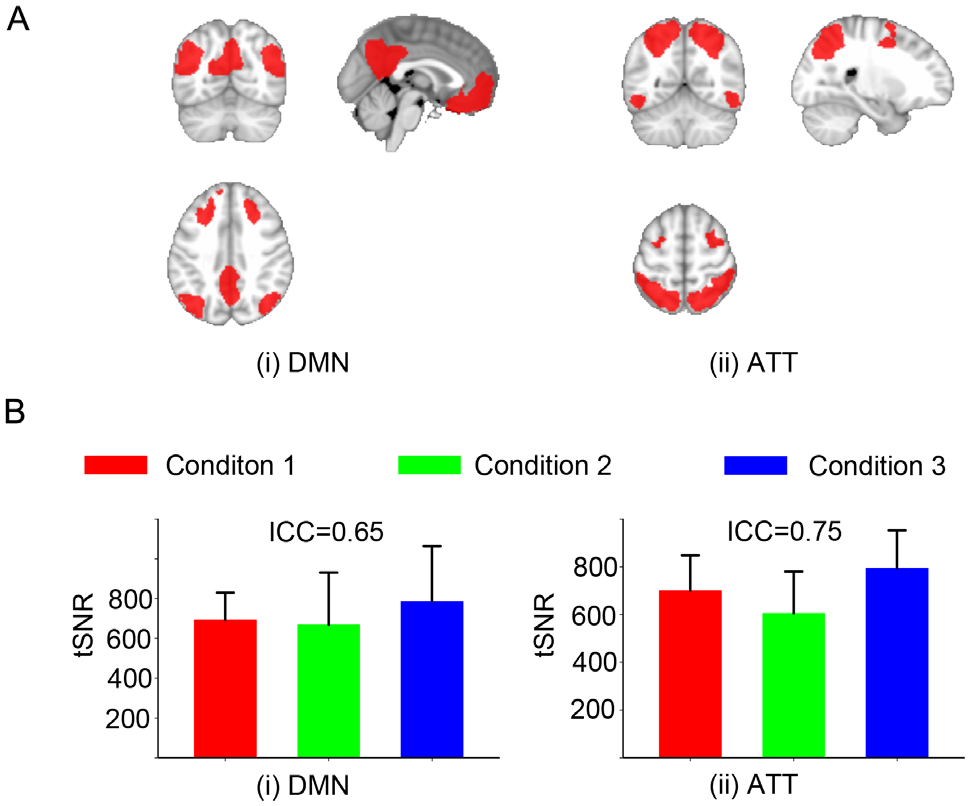
Temporal signal-to-noise ratio (tSNR). Panel A: ROI placements (red regions) in MNI 152 template space, (i) default-mode network (DMN), (ii) attention network (ATT). Panel B: Mean and standard error of tSNR of six subjects across three conditions for default-mode and attention networks. The intra-class correlations are 0.65 and 0.75, respectively.

tSNR was used to estimate temporal stability of measured time course of BOLD weighted echo planar data that can be used for task based studies or for resting state studies. tSNR within regions-of-interest (ROI) were estimated as the ratio of mean signal from all the voxels within the ROI divided by the standard deviation across time [6]. Resting state fMRI identifies the temporal synchronicity between time courses as a measure of connectivity. We explored the tSNR within two functional networks, the default-mode network (DMN) and the attention network (ATT), shown in panel A of Figure 1. For all fMRI BOLD data, the first four volumes were discarded and the remaining volumes were preprocessed, which included the following steps: 1) skull extraction, slice time correction, affine motion correction; 2) spatial smoothing using a Gaussian kernel of full-width at half-maximum 5 mm, and high-pass temporal filtering (150 s); 3) regression of motion correction vectors (3 translations and 3 rotations), mean intensity of white matter, cerebrospinal fluid, and the overall global signal. Temporal SNR was calculated after the BOLD data were preprocessed in the ROIs of the default mode network and the attention network.

### Metric 4: Mean FA and MD Values in ROIs and White Matter for DTI Data

**Figure 2:**
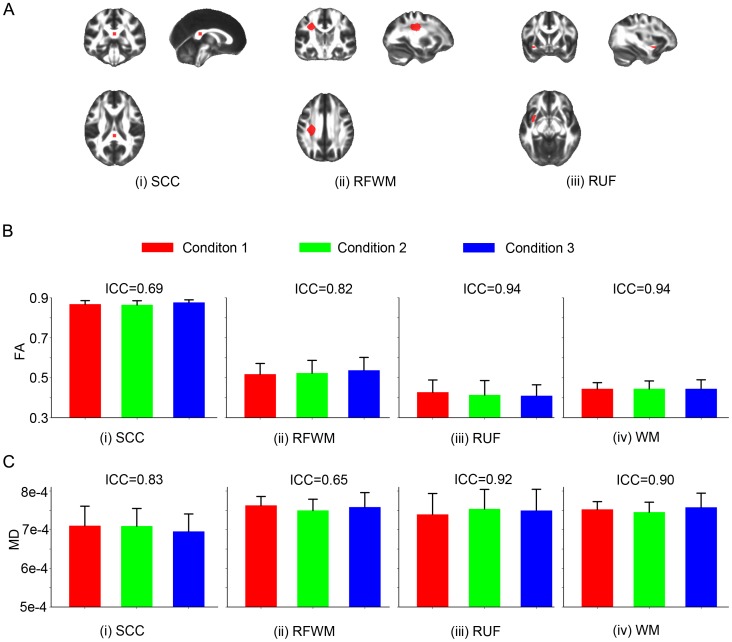
Fractional anisotropy (FA) and mean diffusivity (MD) values of DTI images. Panel A: ROI placements (red regions) in MNI152 template space, (i) Splenium of corpus callosum (SCC), (ii) right frontal white matter (RFWM), and (iii) right uncinate fascicle (RUF). Panel B: Mean and standard error of FA value of six subjects across three conditions for (i) SCC, (ii) RFWM, (iii) RUF, and (iv) WM. The intra-class correlations are 0.69, 0.82, 0.94, and 0.94, respectively. Panel C: Mean and standard error of MD value of six subjects across three conditions for (i) SCC, (ii) RFWM, (iii) RUF, and (iv) WM. The intra-class correlations are 0.83, 0.65, 0.92, and 0.90, respectively.

Analysis for DTI was performed using FMRIB’s Diffusion Toolbox (FDT). DTI images were motion and eddy current corrected and the diffusion tensor was calculated for each voxel. Eigenvalues (λ_1_, λ_2_ and λ_3_) were derived from each tensor, mean diffusivity (MD) was calculated as the average of the three eigenvalues i.e. MD =  (λ_1_+ λ_2_+ λ_3_)/3 and the fractional anisotropy (FA) was calculated as 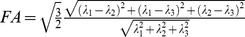. Four ROIs of different scale, the entire white matter, the splenium of the corpus callosum (SCC), the right frontal white matter (RFWM), and the right uncinate fascicle (RUF) (Vollmar et al. 2010), were manually drawn on the study-specific T1 template then transformed to individual subject’s native diffusion space (see panel A of Figure 2). The mean and standard deviation of FA and MD values of six subjects across three conditions for each ROI were calculated.

### Statistical Analysis

The statistical analysis was performed using MATLAB. Two types of statistical assessments were used, one-way ANOVA and intra-class correlation (ICC). One-way ANOVA was used to test group mean differences across three conditions. A statistical threshold of *p<0.05* was considered significant. ICC was used to measure test-retest reliability of the different scanner conditions. ICC is defined as a function of the between-subject variance and the error variance across conditions [7], 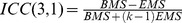, where BMS, EMS and *k* represent between-subject variance, condition error variance, and the number of conditions, respectively. ICC value ranges from 0 to 1. A high ICC value (close to 1) indicates that between subject error dominates the measurement error between different conditions. A small ICC value (close to 0) would indicate that the effect of condition dominates the error.

## Results

### Metric 1

**Figure 3:**
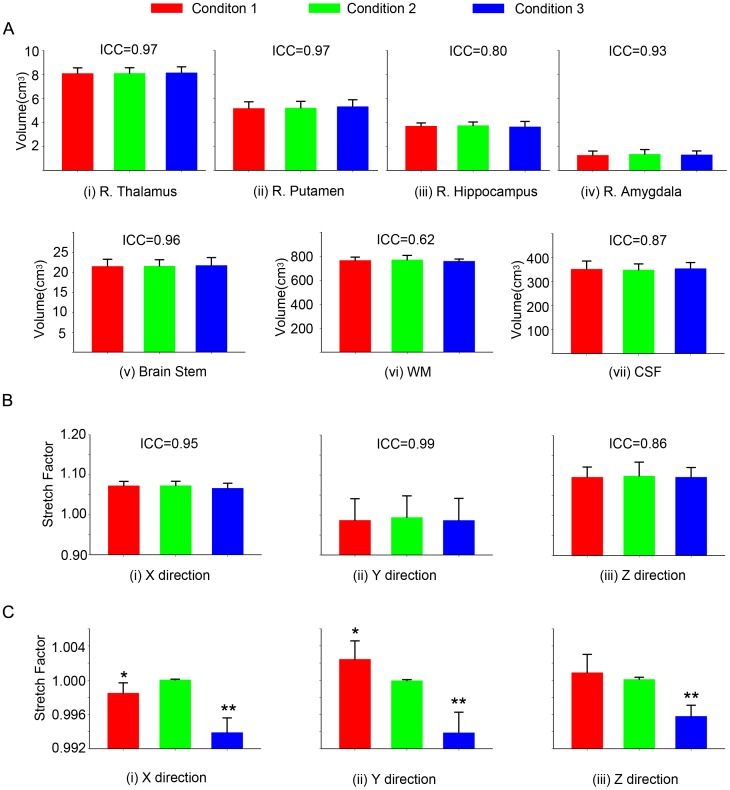
ROI volumes and stretch factor (both based on T1 structure images). Panel A: Mean and standard error of volume (cm^3^) of (i) right Thalamus, (ii) right Putamen, (iii) right Hippocampus, (iv) right Amygdala, (v) Brain Stem, (vi) white matter (WM) and (vii) cerebrospinal fluid (CSF) of six subjects across three conditions. The intra-class correlations are 0.97, 0.97, 0.80, 0.93, 0.96, 0.62 and 0.87, respectively. Panel B: Mean and standard error of stretch factor co-registering to MNI152_T1_1 mm across three conditions in (i) X, (ii) Y and (iii) Z direction. The ICCs are 0.95, 0.99, and 0.86. Panel C: Mean and standard error of stretch factor co-registering to condition 2 T1 image across three conditions in (i) X, (ii) Y and (iii) Z direction. Post-hoc comparisons, for X direction condition 1 (t = 2.93, p = 0.015) and 3 (t = 11.67, p<0.001) groups differ significantly from condition 2 group, indicated by * and **; for Y direction condition 1 (t = 2.78, p = 0.02) and 3 (t = 6.78, p<0.001) groups differ significantly from condition 2, indicated by * and **; for Z direction condition 3 (t = 6.67, p<0.001 ) differs significantly from condition 2, indicated by *. Note that the scale for the stretch factor in panels B and C is different.

T1 (ROIs volume): The average volumes and standard errors for each ROI for all subjects under each condition were plotted in panel A of Figure 3. The exact values and corresponding ANOVA F values as well as ICC for each ROI are reported in Table 2. All ICC values are positive and close to 1 indicating that there are no significant differences between the three conditions compared to the differences between the subjects.

**Table 2:** Average volumes and standard errors for each ROI under three conditions, ANOVA F value, p value, and ICC.

	Con 1(cm^3^)	Con 2(cm^3^)	Con 3(cm^3^)	F_2,15_	p value	ICC
R. Thalamus	8.01±0.47	8.09±0.47	8.15±0.49	0.03	0.97	0.97
R. Putamen	5.17±0.56	5.21±0.55	5.32±0.58	0.11	0.89	0.97
R. Hippocamus	3.70±0.27	3.74±0.29	3.64±0.45	0.15	0.86	0.80
R. Amygdala	1.28±0.24	1.22±0.33	1.42±0.22	0.08	0.93	0.93
Brain Stem	21.47±1.76	21.51±1.60	21.69±1.97	0.03	0.97	0.96
White Matter	764.32±27.8	769.68±36.7	758.08±18.4	0.25	0.78	0.62
CSF	351.88±33.8	348.56±25.4	354.05±25.5	0.057	0.95	0.87

### Metric 2

T1 (stretch factor): Since two templates were used when calculating stretch factor, two sets of stretch factors are presented in panels B (for MNI template) and C (subject specific template from Condition 2) of Figure 3. The exact values and corresponding ANOVA F values as well as ICC for are shown in Table 3 (for MNI template) and Table 4 (for Condition 2 template). There were no significant differences among the three conditions for the stretch factor in any direction when the MNI template was used. However, when each individual subject’s T1 image under condition 2 (scanner 1 post gradient replacement) was used as the template, there were significant differences between the conditions. The post-hoc comparisons, for X direction condition 1 (t = 2.93, p = 0.015) and 3 (t = 11.67, p<0.001) indicate that the conditions 1 and 3 differ significantly from condition 2; for Y direction condition 1 (t = 2.78, p = 0.02) and 3 (t = 6.78, p<0.001 ) groups also differ significantly from condition 2 group; for Z direction condition 3 group (t = 6.67, p<0.001 ) differs significantly from condition 2.

**Table 3:** Average stretch factors and standard errors under three conditions, ANOVA F value, p value, and ICC for MNI template.

	Con 1	Con 2	Con 3	F_2,15_	p value	ICC
X direction	1.073±0.011	1.073±0.011	1.066±0.012	0.62	0.55	0.95
Y direction	0.987±0.054	0.994±0.054	0.987±0.055	0.03	0.97	0.99
Z direction	1.096±0.026	1.099±0.035	1.096±0.024	0.02	0.98	0.86

**Table 4:** Average stretch factors and standard errors under three conditions for individual condition 2 template.

	Con 1	Con 2	Con 3
X direction	0.9985±0.0012	1.0000±0.0001	0.9939±0.0017
Y direction	1.0024±0.0021	1.0000±0.0001	0.9939±0.0024
Z direction	1.0009±0.0021	1.0001±0.0002	0.9958±0.0013

### Metric 3

BOLD (tSNR): The mean and standard error of tSNR across conditions for the 2 network ROIs (DMN and ATT) was (692.2±137.1, 668.3±263.3, 786.6±277.3) and (699.6±147.8, 604.5±175.1, 793.7±158.5), respectively, and are shown in panel B of Figure 1. There are no significant differences across conditions for the DMN (F_2,15_ = 0.43, p = 0.66) and ATT (F_2,15_ = 2.07, p = 0.16). And the ICCs are 0.65 and 0.75, which indicates the measurement error due to the different scanners is small relative to the errors related to the subjects, which demonstrates a consistent tSNR for three scanner conditions.

### Metric 4

DTI (FA and MD) The mean and standard error of FA in the ROIs as well as the ANOVA test, ICC and SCC are shown in the panel B of Figure 2 and Table 5. There are no significant differences across conditions for FA measures. Similarly for MD, the mean and standard error of MD are shown in panel C of Figure 2 and Table 6.

**Table 5:** Average and standard error of FA in the ROIs under three conditions, ANOVA F value, p value, and ICC.

	Con 1	Con 2	Con 3	F_2,15_	p value	ICC
SCC	0.87±0.02	0.86±0.02	0.88±0.01	0.71	0.51	0.69
RFWM	0.52±0.05	0.52±0.06	0.54±0.07	0.15	0.86	0.82
RUF	0.43±0.06	0.41±0.07	0.41±0.06	0.13	0.88	0.94
WM	0.44±0.03	0.44±0.04	0.44±0.05	0.00	1.00	0.94

SCC: Splenium of corpus callosum, RFWM: right frontal white matter and RUF: right uncinate fascicle.

**Table 6:** Average and standard error of MD in the ROIs under three conditions, ANOVA F value, p value, and ICC.

	Con 1(10^−3^)	Con 2(10^−3^)	Con 3(10^−3^)	F_2,15_	p value	ICC
SCC	0.71±0.05	0.71±0.05	0.70±0.05	0.19	0.83	0.83
RFWM	0.76±0.02	0.75±0.03	0.76±0.04	0.28	0.76	0.65
RUF	0.74±0.05	0.75±0.05	0.75±0.06	0.11	0.90	0.92
WM	0.75±0.02	0.75±0.03	.76±0.04	0.28	0.76	0.90

SCC: Splenium of corpus callosum, RFWM: right frontal white matter and RUF: right uncinate fascicle.

## Discussion

We report a battery of metrics used to quantify the reproducibility and image quality for standard neuroimaging data (structural, BOLD and DTI) collected across two identical 3.0 T scanners and following a major hardware repair.

### T1 Anatomic Data

Han et al. [8] and Jovicich et al. [9] examined the scanner upgrade’s (from Sonata to an Avanto) effect on reliability of T1-derived measurements, e.g. cortical thickness and subcortical, ventricular and intracranial brain volumes. The upgrade included replacement of the 1.5T magnet, the RF and gradient system, and software. They showed that pooling data across the scanner upgrade did not degrade the measurement reproducibility. Recently Kruggel et al. [10] examined impact of scanner hardware and imaging protocol on image quality and segmented compartments (CSF, GM and WM) volume precision in the ADNI cohort. Image quality was rated by the Signal-to-Noise ratio (SNR), higher SNR corresponds to better image quality. They reported that although SNR is dependent on hardware, software, environmental and subject parameters, the SNR is mostly dependent on the scanner hardware and explained up to 74% of variance. For compartment volumes, they reported the precision of repeated scans of the same subject on different scanners with different field strengths and vendors is much worse than that on the same scanner over time.

In the present study, we focused on the differences between two identical scanners sited the same way and the effect of a major repair (gradient coil replacement) on morphometry measures (brain volumes and stretch factor). Our results show the metrics proposed are highly reproducible for volumetric data and when the data are pooled to a standard template. However when using a study specific template, the native anatomic data were sensitive to geometric distortions introduced by the gradient replacement and between different scanners. It is not surprising that differences between same scanner before and after hardware repair were smaller than differences between the two different scanners. Note that these differences in scale are far less than 1% and did not alter volumetric measures in the ROIs tested.

### BOLD

For task-based fMRI studies, a diverse collection of methods have been used to assess fMRI task-induced activation reliability (see review by Bennett et al. [11], including activated cluster overlap and Intra-class correlation). For resting-state fMRI, the test-retest variability of the resting state network has been examined by Shehzad et al. [12] using seed-based resting state networks and by Zuo et al. [13] using ICA-based networks. Temporal Signal-to-Noise Ratio (tSNR) is an important factor influencing fMRI reliability in both task and resting state studies that represents an overall measure of image quality. In the case of resting state fMRI, the best way to quantify “signal” and “noise” is not clear and depends on the method of data analysis. We propose the use of tSNR to indicate temporal stability of the BOLD data for both task and resting state analysis. The rationale for using two resting state networks as ROIs for calculating tSNR instead of anatomical ROIs is that signals within the same functional network exhibit similar/coherent spontaneous fluctuations and registration issues while small anatomical ROIs may introduce additional variation which can be large enough to obscure the scanner induced variations. Default-mode and attention networks were selected for their reliable and complementary nature [14], [15].

Our results showed that the measurement error caused by different scanners is small relative to noise introduced by different subjects. This demonstrates that the BOLD data were not sensitive to the differences between conditions using the tSNR in resting state networks. A potential metric for resting state data is the ratio of connectivity strength within a known network divided by the connectivity strength between a functional irrelevant network. However unlike T1 and DTI measures, the strength of the connectivity heavily depends on the physiological/cognitive status of the subject during the data collection, therefore it may not be a suitable metric for testing reliability of the imaging hardware and environmental changes.

### DTI

Using ROI-based analysis, Vollmar et al. [16] showed that with two identical 3T scanners there was a consistently low variation of FA measures between scans for both intra- and inter-site rescanning. Using a similar ROI-based analysis, we showed that the FA values are highly reproducible likely due to the close proximately so that they share the same physical environment and resources as well as the identical installation design. In contrast, using whole brain Tract-Based Spatial Statistics (TBSS) and voxel-based analysis, Takao et al. [17] showed inter-scanner variability and a software upgrade (not hardware upgrade) introduced a significant bias on longitudinal (1 year) FA comparisons. The difficulty in interpreting Takao’s data is that these changes could be real or introduced by the software. Any sort of adjustment made by the service engineer during a software upgrade or tune-up could alter these gradient demanding data. In our study we measured the different conditions over a short two week period to avoid these complications.

Following Vollmar et al. [16], whole brain white matter, SCC, RFWM, and RUF were chosen in the current study as they represented four kinds of fiber characteristics in white matter: the fibers are mainly parallel and densely-packed in SCC, orientation-crossed in RFWM, and small in RUF. The ROI representing SCC was defined carefully in the standard template as shown in panel A (i) of Figure 2 to make sure after projecting back to the native space of each subject it remained only in the WM regions. The mean FA values of the aforementioned three ROIs and whole brain white matter were comparable but in general lower than those reported by Vollmar et al., which might be due to the differences in the ROI selection, scanner type, field strength, coils, the number of directions, data analysis and individual differences. At all scales of assessment, our data did not find any differences across scanners or following the gradient coil replacement for either FA or MD.

### Conclusion

We achieved consistent measures for T1 brain structure volumes, stretch factor, tSNR for resting state BOLD signals and mean FA/MD values in specific ROIs and the whole brain between two identical 3T scanners, and before and after gradient coil replacement. These findings provide statistical validations for conducting longitudinal work on multiple systems that are identical. Furthermore, even a major hardware repair did not alter the metrics for all three types of neuroimaging data. It is important that the proposed metrics be used for routine quality control for all data collected to identify any issues related to hardware, software or subjects. Finally, in order to use these metrics in a multi-center study, one would have to employ the “human phantom” approach resulting in someone travelling to all of the sites. A traveling phantom may be appropriate for short term multi-center trials but not ideal for longitudinal studies as changes are expected in the “human phantom” that could complicate the interpretation of the results.
